# Racial/Ethnic Inequities in Paid Parental Leave Access

**DOI:** 10.1089/heq.2021.0001

**Published:** 2021-10-13

**Authors:** Julia M. Goodman, Connor Williams, William H. Dow

**Affiliations:** ^1^ Oregon Health and Science University–Portland State University School of Public Health, Portland, Oregon, USA.; ^2^ University of California–Berkeley School of Public Health, Berkeley, California, USA.

**Keywords:** parental leave, racial/ethnic inequities, California, workers

## Abstract

**Purpose:** Severe racial inequities in maternal and infant health in the United States are caused by the many forms of systemic racism. One manifestation of systemic racism that has received little attention is access to paid parental leave. The aim of this article is to characterize racial/ethnic inequities in access to paid leave after the birth of a child.

**Methods:** We analyzed data on women who were employed during pregnancy (*n*=908) from the Bay Area Parental Leave Study of Mothers, a survey of mothers who gave birth in the San Francisco Bay Area in 2016–2017. We examined differences in access to government- and employer-paid leave, the duration of leave taken, and the percent of usual pay received while on leave. To explore these differences, we further examined knowledge of paid leave benefits and sources of information.

**Results:** Non-Hispanic (NH) black and Hispanic women had significantly less access to paid leave through their employers or through government programs than their NH white and Asian counterparts. Relative to white women, Asian, Hispanic, and black women received 0.9 (*p*<0.05), 2.0 (*p*<0.01), and 3.6 (*p*<0.01) fewer weeks, respectively, of full-pay equivalent pay during their parental leaves. Despite inequitable access to paid leave, the duration of parental leave taken did not differ by race/ethnicity.

**Conclusions:** Inequitable access to paid parental leave through both employers and government programs exacerbates racial inequities at birth. This form of structural racism could be addressed by policies expanding access to paid leave.

## Introduction

Striking racial inequities in maternal and infant health in the United States are well documented. The rate of severe maternal morbidity, or life-threatening pregnancy complications, has increased significantly over recent decades for all racial/ethnic groups, but remains consistently higher for non-Hispanic (NH) black, Hispanic, and Asian/Pacific Islander women compared to NH white women, even after adjusting for covariates.^1^ NH black women, in particular, are more than three times as likely to die from pregnancy-related causes than NH white women^2^ and face significantly higher risks of preterm birth and other adverse birth outcomes.^3^

The infant mortality rate among NH black infants is more than twice as high as that among NH white infants.^4^ Black infants are significantly less likely to initiate breastfeeding and, among those who do, are less likely to be breastfeed for at least 6 months.^5^

These inequities are thought to be caused by numerous factors, including systemic and interpersonal racism.^6,7^ Structural racism, which operates at the macrolevel and influences systems, ideologies, social forces, and institutions, has been identified as the root cause of a range of health inequities.^8^ Allostatic load, or the cumulative physiological effects of stress over the life course, has gained traction as a theory to explain racial inequities in pregnancy outcomes due to the burden of racism experienced by women of color.^9^

In addition, structural racism in health care leads to black women often receiving poorer quality care relative to white women. For example, residential segregation rooted in racism leads to black women being more likely to deliver at poor quality hospitals^10^ and black patients' reports of pain being taken less seriously than those of white patients.^11^ Women who experience perceived discrimination during prenatal visits, including discrimination based on their race or ethnicity, have more than twice the odds of nonattendance at postpartum visits,^12^ which can contribute to increased risk for maternal mortality.

Another manifestation of structural racism that has received less attention is differential access to paid parental leave. Substantial research documents beneficial effects of parental leave-taking for both new parents and their children.^13–22^ Paid parental leave, which enables workers to take time away from work to recover from childbirth and care for a new baby, is associated with decreased low-birth-weight births and infant mortality, increased breastfeeding, and improved maternal mental health.^19–26^ In 2018, the American College of Obstetricians and Gynecologists (ACOG) endorsed at least 6 weeks of fully paid leave for all new mothers to reduce high rates of maternal mortality in the United States.^27,28^

Paid leave confers economic as well as health benefits. Availability of paid leave increases household income, decreases risk of poverty, and reduces some forms of material hardship, especially among less-educated and low-income single mothers.^29,30^ (This is in addition to the fact that access to paid leave itself is also a marker of the underlying racial segregation of labor markets that also directly contribute to racial differences in wages, as discussed further below.) Importantly, these health and labor market benefits are largely associated with paid, but not unpaid, leave.^16^

The small body of work examining racial/ethnic differences in the impact of paid leave policies on economic and health outcomes has found mixed results. For example, Rossin-Slater et al. found that, while California's paid family leave (CA-PFL) program increased leave-taking for all mothers, the largest absolute gain in leave-taking was among black mothers who, as a group, had the lowest baseline levels of maternity leave-taking.^31^ This is reinforced by work from Joshi and colleagues showing that black and Hispanic parents are less likely than their white counterparts to be eligible for and potentially able to afford unpaid leave.^32^

Other reports show similar eligibility for unpaid leave across racial groups, yet higher unmet need for leave among black workers relative to white and Asian workers, suggesting that eligibility for job-protected leave alone potentially masks financial or other limitations on workers' ability to take leave.^33^ Simulations of leave-taking under various policy scenarios suggest that paid family and medical leave (PFML) (vs. unpaid leave) would enable black workers to gain a greater percentage of their family income back relative to white workers, but would have a relatively smaller impact on preventing short-term economic hardship.^34^

In terms of health outcomes, Hamad et al. examined paid family leave policies in California and New Jersey and found that, relative to white women, black women had reduced breastfeeding at 12 months, while Hispanic women had increased exclusive breastfeeding at 6 months.^20^ Recent studies of infant immunizations^35^ and birth outcomes^36^ found no racial differences, while others report that small subgroup sample sizes prevented detection or examination of racial/ethnic differences.^22,24^

The lack of a national PFML program and, instead, reliance on a patchwork of state and local policies that provide partial pay for some workers mean that many of the most economically vulnerable parents lack access to paid leave. The Family and Medical Leave Act provides job-protected, unpaid caregiving leave to covered workers, but strict eligibility requirements exclude about half of the workforce.^37^

Access to paid leave, which financially enables many lower income workers to take advantage of leave benefits, is even more limited: just eight states (CA, RI, NJ, NY, WA, MA, CT, and OR) and the District of Columbia have government PFML programs.^38^ Only about 19% of U.S. workers have any paid family leave offered through their employers, with even lower access among part-time workers, nonunionized workers, workers in low-wage industries, and workers at small firms.^39^

Surprisingly, little research has examined racial and ethnic inequities in access to paid leave. These inequities could stem from occupational segregation affecting the likelihood of working for a firm that offers paid leave and job characteristics, such as working full versus part time and job tenure, which could impact eligibility for employer- and government-paid leave benefits.

Two analyses draw on data from a 2011 Leave Module in the Bureau of Labor Statistics' American Time Use Survey that asked whether respondents received any paid leave from their employer and whether they were able to take paid leave for the birth or adoption of a child, for their own medical condition, to care for a family member, or for other reasons. These analyses show that just 23–25% of Hispanic parents have access to paid parental leave, compared to 47–50% of NH white and 41–43% of NH black parents.^40,41^

This unequal access to paid parental leave does not appear to be due to differences in the likelihood of working full time: Hispanic workers are equally likely to be employed full versus part time compared to NH workers.^40^ Differential access more likely stems from the types of jobs workers hold. Hispanic workers are most likely to fall in the lowest wage brackets^40^ and, relative to white and Asian workers, both NH black and Hispanic workers are underrepresented in professional-class jobs^42^—jobs that are more likely to provide benefits like paid leave.^40^

Once demographic and employment characteristics are controlled for, the differences in access to paid parental leave between NH whites and blacks go away; however, Hispanic workers remain significantly less likely to receive these benefits.^41^ Decomposition of these differences in paid leave access points to the importance of immigration and citizenship status, as well as differential returns to full-time employment for Hispanic workers.^41^

This also suggests additional mechanisms, such as potentially a lack of awareness of available benefits or lack of adequate support from employers to facilitate leave-taking. In a survey of California workers who had recently become a parent or had a close family member become seriously ill, less than half of respondents were aware of the state's Paid Family Leave program, and awareness was lowest among low-wage workers (38%), immigrants (34%), Latinx workers (34%), and workers with less than a high school diploma (21%).^43^

Workers of color may also be less likely to work in jobs that are covered by PFML policies, where applicable. For example, most public sector (i.e., governmental) workers are excluded from paid family leave programs in California, Rhode Island, New York, District of Columbia, and Connecticut^38^ (although some states, including California, allow public sector workers to opt in) and black parents are overrepresented in public sector jobs.^42^ In addition, all state PFML programs have some employee eligibility requirements that specify minimum earnings and/or hours worked in the base period, which may disproportionately exclude those in part-time, seasonal, or low-wage jobs—jobs in which women of color are overrepresented.^33,42^

Importantly, the amount of leave actually taken after giving birth does not appear to vary dramatically across racial/ethnic groups, and recent studies have shown inconsistent leave-taking patterns.^41,44–46^ If parents take the same amount of leave regardless of pay, this implies that non-white parents are less likely to receive financial support in the newborn period—parents who already own far less wealth than white parents as the result of racist social and economic policies, racial segregation, and slavery.^47^

Even among workers who receive some paid leave, the amount and duration of pay may vary. While employer-provided paid leave often provides full pay for a set number of weeks, state PFML programs offer a percentage of usual wages.^38^ The consequence is that workers relying exclusively on state PFML programs may face a more severe financial penalty for taking leave relative to workers receiving fully paid leave through their employers. The extent to which this impacts workers, however, remains unknown as most existing data sources do not differentiate between government- and employer-provided pay, nor do they assess the proportion of usual pay received while on leave. This gap in the literature leaves the full impact of inequitable access to paid leave unknown.

This study draws upon a unique data source that includes detailed questions on the amount and source(s) of pay available after the birth of a child to better understand racial/ethnic inequities in paid leave access.

The objective of our study was to examine whether there are racial/ethnic inequities in (1) access to government- and employer-paid leave; (2) amount of pay received while on leave; and (3) the duration of leave taken after the birth of a child. To better understand these inequities, we also examined differences in knowledge of paid leave benefits and sources of information. Finally, to summarize the magnitude of the inequity in paid leave access, we calculated racial/ethnic disparities in the average number of full-pay equivalent (FPE) weeks of paid leave taken.

We present all our results without controlling for covariates to avoid concealing the influence of important sociodemographic and job characteristics that may lead to differential access and utilization of paid leave benefits.

## Methods

### Data

We use data from the Bay Area Parental Leave Study of Mothers, a survey of mothers who gave birth in the San Francisco Bay Area in 2016–2017. We mailed invitations to a stratified random sample from birth records for all women who delivered live births in the San Francisco Bay Area in 2016 or 2017 to participate in a 25-min online survey. Women who were employed or whose partners were employed during pregnancy, whose child was still living with them, and who could complete the survey in English or Spanish were eligible to participate. Participants were offered a $15 gift card for their participation. Nonresponders were mailed up to two reminder cards (the second with a higher incentive offer); the subset of remaining nonrespondents with identifiable phone numbers was subsequently called.

We completed data collection for 2016 births in August 2018 and for 2017 births in January 2019. One thousand three hundred-four women completed our survey (20.4% response rate). Our analytic sample for this article includes respondents who were employed by someone else during pregnancy (i.e., excluding nonemployed and self-employed women) and whose self-reported race was NH white (hereafter referred to as white), NH Asian (hereafter referred to as Asian), NH black (hereafter referred to as black), or Hispanic of any race (hereafter referred to as Hispanic) (*N*=908).

The Bay Area Parental Leave Study of Mothers was originally conducted to examine the impact of the San Francisco Paid Parental Leave Ordinance (PPLO), a paid leave policy that went into effect for San Francisco employers in 2017, on leave-taking among new mothers. Previous analyses showed that the PPLO had no impact on leave-taking among women in the Bay Area.^48^ Thus, for this analysis of access to paid leave, we combine pre- and post-PPLO waves of data and all employed women, regardless of county of employment.

### Measures

Our primary independent variable is mothers' self-reported race/ethnicity. We restrict to women who identified as white, Asian, black, or Hispanic (we drop 6.6% of our sample who reported their race as “other” or who had missing race/ethnicity).

Employed women were asked a series of questions about their job(s) during pregnancy and whether and how much leave from work they took after the birth of their child. All respondents took some postnatal parental leave, so our analyses focus on duration only. We assessed the duration of postnatal parental leave among women who returned or planned to return to their same job with the question, “How old was your child when you returned to working for pay, even part time?” Women who had not yet returned to work, but who planned to were categorized as taking 12 or more weeks of leave, as all interviews took place greater than 12 weeks postpartum.

We assessed paid leave access using four measures:

(1) we asked women the following question: “Thinking about all sources of pay during your leave, was the amount of money you received equal to your regular pay?” Women who said no were asked to report the percent of total pay on average they received while on leave. Combining these two questions, we categorized the percent of usual pay as no pay, less than 50% of regular pay, 50–99% of regular pay, and 100% of regular pay.

(2) We categorized employer-provided paid leave based on whether the respondent reported that their employer provided paid leave and, if so, for how many weeks: no paid leave, 1 to 5 weeks, 6 to 11 weeks, and 12 or more weeks.

(3) We examined government-provided paid leave, which, in California, takes the form of State Disability Insurance and Paid Family Leave and together provide 12 weeks (14 weeks for Cesarean delivery) of paid leave for parents recovering from childbirth. We therefore made the assumption that women who reported receiving any government-provided paid leave and who took at least 12 weeks (14 for Cesarean delivery) of leave received paid leave from the government for 12 or 14 weeks. For women who reported receiving government-provided paid leave and who took less than 12 weeks of leave, we assumed that they received paid leave from the government for the duration of their leave. We therefore categorized government-paid leave as no paid leave, 1 to 11 weeks, or 12 or more weeks. Note that these programs do not cover public sector (i.e., government) employees unless their employer opted in. We therefore do not know whether individual employees, particularly those who are employed in the public sector, are eligible for paid leave through these programs.

(4) We multiplied the percent of usual pay during leave by the number of weeks of leave taken to estimate the FPE weeks of paid leave. For respondents whose percent of usual pay was provided as a range, we used the midpoint (e.g., “less than 50%” was assigned 25% and “51–75%” was assigned 63%). As a sensitivity analysis, we assigned those reporting “less than 50%” first no pay and then 49% pay.

Additional outcomes of interest included whether respondents understood the maternity leave benefits available to them and whether their employer or anyone else was a source of information about their maternity leave benefits.

We present demographic and job characteristics by race/ethnicity to inform interpretation of our results. Job characteristics include public versus private sector, job tenure, hours worked during pregnancy, employer size, and employer location; sociodemographic characteristics include marital/partner status, education, language spoken at home, and Medicaid coverage during pregnancy as a proxy for household income.

### Approach

We used unadjusted linear probability models and ordinary least squares models to examine differences in leave-taking probability and duration across white, black, Asian, and Hispanic women. We reported robust standard errors and incorporated probability weights to account for nonresponse and oversampling of San Francisco residents, Spanish speakers (proxied by mother's immigration from a Spanish-speaking country), and low-income (proxied by Medicaid status) women.

Racial/ethnic disparities in access to paid leave could result from differences in “downstream” characteristics—demographic or human capital differences and geographic and occupational segregation—that are produced by “upstream” processes of discrimination (e.g., inequitable access to education, hiring discrimination, and historical redlining practices). Controlling for these characteristics would conceal potential consequences of racism that are important to capture. Therefore, we present unadjusted models, with sensitivity analyses focusing on narrower subpopulations (e.g., full-time workers with at least 6 months job tenure; private sector workers) to interrogate observed disparities.

All analyses were conducted in Stata version 14.2 (College Station, TX: StataCorp LLC). Study procedures were approved by the California Health and Human Services Agency's Committee for the Protection of Human Subjects.

## Results

Table 1 reports demographic and job characteristics of our sample of Bay Area mothers, grouped by race/ethnicity. The racial/ethnic composition of our sample is representative of mothers of young children in the Bay Area, with a distribution similar to that in the American Community Survey (ACS) during our study years (5.9% black, 18.5% Hispanic, 31.2% Asian, and 44.4% white in our sample compared to 4.5% black, 12.8% Hispanic, 35.9% Asian, and 44.2% white in ACS). For each characteristic, we report *p*-values from chi-squared tests.

**Table 1.** Descriptive Statistics

	White (44.4%)		Asian (31.2%)		Black (5.9%)		Hispanic (18.5%)		Chi-squared
	*n*	%	*n*	%	*n*	%	*n*	%	*p*
Marital status									
Married or living as married	372	97.3	263	96.3	39	65.6	146	84.8	<0.001
Single, separated, divorced, widowed	11	2.8	14	3.7	24	34.4	31	15.2	
Education									
High school/GED or less	5	1.9	14	3.9	19	23.5	54	33.6	<0.001
Some college	30	7.7	34	12.4	23	43.5	67	30.0	
College graduate	349	90.4	229	83.7	20	33.0	56	36.5	
Other household children <5									
None	248	66.3	168	61.7	37	56.9	94	51.8	0.052
1 or more	128	33.7	100	38.3	24	43.1	76	48.2	
Language spoken at home									
English	356	92.1	168	62.5	58	93.0	116	58.6	<0.001
Other	28	7.9	110	37.5	5	7.0	61	41.4	
Nativity									
U.S. born	53	85.2	166	43.1	6	89.3	72	56.9	<0.001
Foreign born	333	14.8	115	56.9	57	10.7	106	43.1	
Insurance during pregnancy									
Medi-Cal	22	4.0	46	12.7	37	45.4	109	49.5	<0.001
Private/Other/Uninsured	364	96.0	235	87.4	26	54.6	69	50.5	
Job type									
Private for profit	240	62.7	195	69.5	34	59.0	124	65.1	0.316
Private nonprofit	81	20.1	41	14.5	9	12.5	28	16.2	
Public employee	65	17.2	45	16.1	20	28.5	26	18.7	
Job tenure									
Less than 6 months	16	4.7	13	5.0	9	15.3	19	12.9	<0.01
Between 6 and 11 months	30	8.0	38	12.2	13	17.1	29	17.8	
Between 1 and 4 years	189	48.6	138	50.3	29	48.2	88	45.8	
5 or more years	146	38.6	91	32.5	10	19.3	39	23.5	
Hours per week									
Less than 8	3	0.9	7	2.0	2	3.7	13	6.3	<0.001
Between 8 and 23	26	6.5	25	7.9	10	12.5	22	12.7	
Between 24 and 35	41	10.5	37	11.9	18	32.1	45	22.7	
More than 35	312	82.1	210	78.2	32	51.7	95	58.3	
Work schedule									
Regular daytime	325	85.0	229	84.2	45	69.6	131	74.3	<0.01
Regular evening/night shift	13	3.5	14	4.7	6	5.8	22	12.1	
Variable schedule	46	11.6	36	11.1	12	24.5	25	13.6	
Employer size									
<20	40	11.0	49	17.4	12	23.8	41	20.4	<0.001
20–49	35	8.7	19	6.5	10	8.8	37	21.4	
50–99	26	6.5	17	5.5	6	10.6	18	9.3	
100–199	31	8.5	20	7.1	8	14.8	13	7.8	
200–499	41	10.8	23	8.6	9	13.9	12	6.3	
500 or more	212	54.5	152	55.0	18	28.1	53	34.8	
Employer location									
San Francisco	198	50.0	143	47.0	33	50.9	64	31.6	<0.01
Other Bay Area	187	50.0	137	53.0	30	49.1	110	68.4	
Duration of leave									
<6 weeks	11	3.3	13	5.0	5	5.8	10	8.8	0.1692
6–11 weeks	36	11.2	39	15.0	6	7.7	20	9.8	
12+ weeks	285	85.5	198	80.1	43	86.5	114	81.4	
Percent of usual pay received									
<50%	44	12.7	51	20.7	20	29.6	44	33.0	<0.001
50–99%	184	53.0	113	46.2	28	52.9	60	34.9	
100%	120	34.3	85	33.2	10	17.5	40	32.1	
Government pay duration									
None	64	19.3	51	23	22	46.6	39	36.2	<0.001
1–11 weeks	28	9.6	33	12.5	3	4.2	20	12.3	
12–14 weeks	236	71.2	152	64.5	28	49.3	81	51.6	
Employer pay duration									
None	152	45.0	119	48.7	40	68.3	89	60.3	<0.05
1–5 weeks	21	5.8	16	6.4	0	0.0	13	6.6	
6–11 weeks	60	18.8	49	19.0	8	18.7	22	16.1	
12+ weeks	107	30.3	67	25.9	9	13.0	24	17.0	

The *p*-values in this table represent results from weighted chi-squared tests. (Because of the complex sample design, the Pearson chi square was transformed to an *F* statistic with approximate degrees of freedom to find the *p*-value.)

GED, General Education Development.

We observe that most demographic characteristics and all job characteristics vary by race. Black mothers are least likely to be married or cohabiting. Large majorities of white and Asian mothers have a college degree (90.4% of whites and 83.7% of Asians), whereas 33% of black mothers and 36.5% of Hispanic mothers have graduated college. Asian and Hispanic mothers are both much more likely than their counterparts to report speaking a language other than English in the home (37.5% and 41.4%, respectively), and to be born outside of the U.S. Hispanic and black mothers are significantly more likely to be insured by Medi-Cal (California's Medicaid program; 49.5% and 45.4%, respectively) relative to white (4.0%) and Asian (12.7%) women. The number of household children younger than five years does not appear to be associated with race or ethnicity in our sample.

Job characteristics also show strong associations with race/ethnicity among Bay Area mothers. Modest, but significant differences exist in job tenure, with white and Asian mothers being more likely to have worked in their job for at least 5 years and less likely to have a job tenure less than 6 months than black and Hispanic mothers. Similarly, black and Hispanic mothers are less likely to work full time, with only 51.7% of black and 58.3% of Hispanic mothers working over 35 h per week (compared to 82.1% of white mothers, *p*<0.001).

We observed a similar pattern for size of employer, with a majority of white and Asian mothers working for large (>500 employees) firms, compared to only 28.1% and 34.8% of black and Hispanic women, respectively. White, Asian, and black women work in jobs that are distributed roughly equally between San Francisco and the rest of the Bay Area, but only 31.6% of Hispanic women work in San Francisco. While racial/ethnic differences in job type were not statistically significant, there is a trend toward black women being overrepresented in public sector jobs and underrepresented in private for-profit jobs. We comment on the distribution of our dependent variables by race below.

**FIG. 1.**
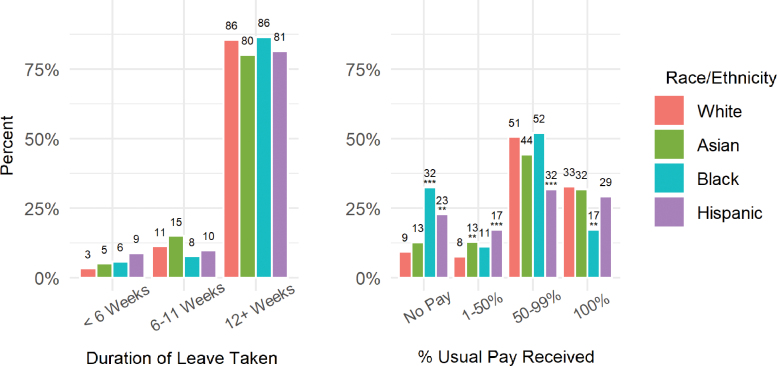
Duration of maternity leave taken and percent of usual pay received, by race/ethnicity. **p<0.05; ***p<0.01. Asterisk show significance relative to whites.

Given these differences in job characteristics, black and Hispanic women may be less likely to be eligible for paid leave programs. In fact, when we examined whether and how much pay women received while on leave, strong racial/ethnic disparities emerged (Fig. 1). Relatively few white (10%) and Asian (13%) women received no pay while on leave, whereas significantly more black (33%; *p*<0.01) and Hispanic women (25%; *p*<0.05) received no pay. By contrast, only 10% of black women received full pay while on leave, relative to about one-third of white women (*p*<0.05).

Despite these differences in access to paid leave, the duration of leave taken was similar across all racial/ethnic groups (Fig. 1). The majority of women returned to work when their new baby was at least 12 weeks old; very few returned before 6 weeks. We similarly found no significant difference in the likelihood of taking more than 16 weeks (Supplementary Fig. S1).

**FIG. 2.**
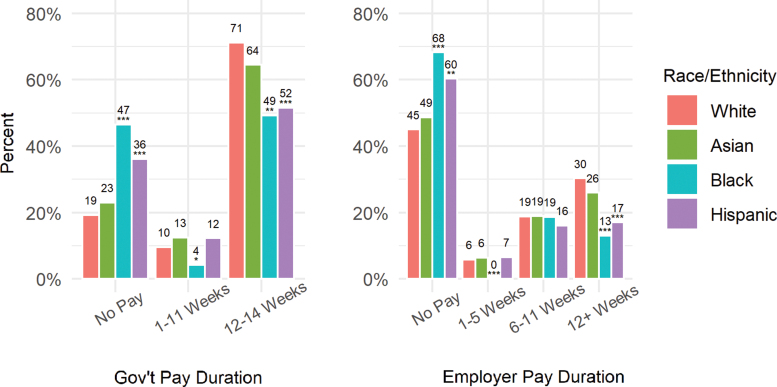
Duration of government- and employer-paid leave, by race/ethnicity. *p<0.10; **p<0.05; ***p<0.01. Asterisk show significance relative to whites.

Lower pay among black and Hispanic women appears to be due to lower likelihood of receiving pay both from the government (e.g., CA-PFL or State Disability Insurance programs) and from employers (Fig. 2). While 71% of white women received 12 to 14 weeks (depending on their mode of delivery) of pay from the government, just about half of black (49%; *p*<0.05) and Hispanic (52%; *p*<0.01) women received that much pay. In contrast, 47% of black and 36% of Hispanic women did not receive any pay from the government, compared to just 19% of white women (*p*<0.01).

Similarly, while a majority of white women received at least some pay from their employers, 68% of black (*p*<0.01) and 60% of Hispanic (*p*<0.05) women did not receive any pay from their employers. Both groups were significantly less likely to receive at least 12 weeks of paid leave from their employers compared to white women.

Among Hispanic women, these disparities persisted even when restricting our analysis to women employed full time (>35 h per week) and who had been employed for at least 6 months (although differences were no longer significance at 5% level due to smaller sample size) (Supplementary Fig. S2). Black women who had been employed full time for at least 6 months were also significantly less likely to receive any pay from their employer relative to their white peers; however, this subgroup of workers was not significantly less likely to receive government pay during their maternity leave relative to white women.

Employment sector did not explain these differences: disparities in access to employer-paid leave remained for both black and Hispanic women when restricting to those employed by private employers (Supplementary Fig. S3) and private for-profit employers (Supplementary Fig. S4).

**FIG. 3.**
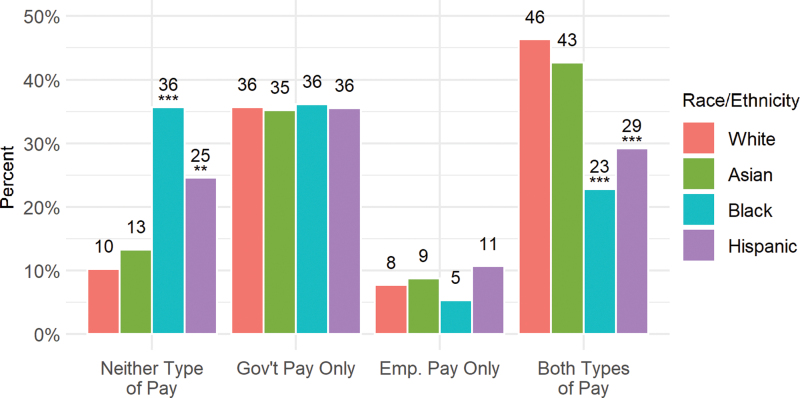
Type of pay received, by race/ethnicity. **p<0.05; ***p<0.01. Asterisk show significance relative to whites.

Bringing these sources of pay together, we find that almost half of white women (and 43% of Asian women) received both government and employer pay during their leave, significantly more than either black (23%; *p*<0.01) or Hispanic women (29%; *p*<0.01) (Fig. 3). In contrast, black and Hispanic women were significantly more likely than white women to report not receiving either type of pay (36% and 25%, respectively). Interestingly, 35–36% of women reported government pay alone across all four racial/ethnic groups.

**FIG. 4.**
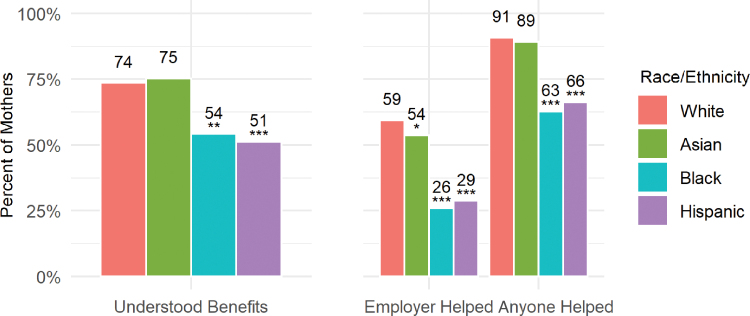
Percent of mothers who understood their maternity leave benefits and sources of information about benefits, by race/ethnicity. *p<0.10; **p<0.05; ***p<0.01. Asterisk show significance relative to whites.

To help understand why we observed racial/ethnic disparities in reported access to paid leave benefits, we examined mothers' knowledge of their maternity leave benefits and the main sources of information about these benefits (Fig. 4). While about three-quarters of white and Asian women reported that they understood the maternity leave benefits available to them, significantly fewer black (54%; *p*<0.05) and Hispanic (51%; *p*<0.01) women did.

This likely reflects differential access to information. Black and Hispanic women were significantly less likely than white women to report that their employer helped them learn about their maternity leave benefits (−33 and −31 percentage points, respectively; *p*<0.01). In fact, just 63% of black women and 66% of Hispanic women reported that they received help from anyone, compared to 91% of white women (*p*<0.01).

**FIG. 5.**
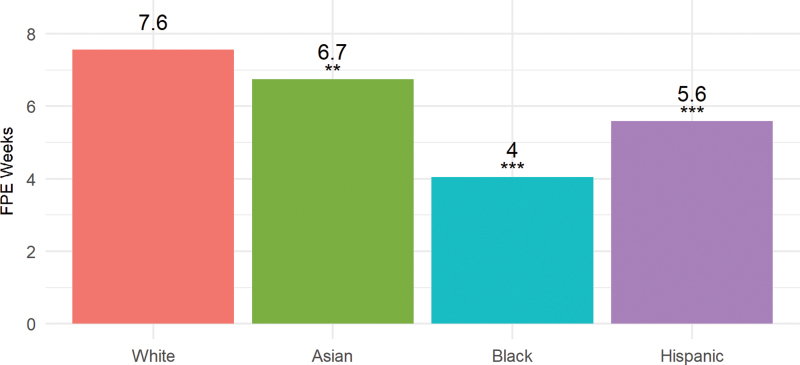
FPE, by race/ethnicity. FPE, full-pay equivalent weeks of paid leave. **p<0.05; ***p<0.01. Asterisk show significance relative to whites.

Putting wage replacement level together with the duration of paid leave offered, we developed a measure of FPE weeks of paid leave. White women, on average, received 7.6 FPE weeks of paid leave (Fig. 5). Asian women received slightly less (6.7 FPE weeks). Again, black and Hispanic women received significantly less paid leave: 4.0 and 5.6 FPE weeks, respectively.

## Discussion

Consistent with prior research, we find that the duration of leave taken after the birth of a child was similar across racial/ethnic groups; however, the economic implications of that leave differ significantly. We found that black women on average receive 3.6 fewer FPE weeks of paid leave than white women. To quantify that, consider a woman working full time at a minimum wage of $15: in that case, 3.6 fewer weeks of pay amounts to $2160 less over the course of her leave—a large sum for these women.

To put this in perspective, a substantial literature links increased income such as from the earned income tax credit (EITC) with improvements in maternal and infant health.^49^ One study finds that an additional $500 per year in EITC reduces the number of bad mental health days by 19% among mothers^50^; another finds that an additional $500 from a similar Canadian program decreased depression scores by 11% among mothers with a high school degree or less.^51^ This magnitude of extra EITC income has also been found to improve birth outcomes,^52^ as well as child development.^53^ The accumulated evidence suggests that disparities in access to paid parental leave are a preventable source of racial inequities in health that begin at birth.

The reasons underlying disparities in paid leave access are not entirely clear. One hypothesis is that black and Hispanic women are less likely to hold jobs with eligibility for employer- and government-paid leave due to occupational segregation and policy choices that have been made to benefit professional, salaried workers. Part-time workers, for example, are less likely to be eligible for paid leave benefits,^39^ and in our sample, black and Hispanic women are overrepresented in part-time work. Unlike many employer policies, CA-PFL program has generous eligibility in terms of hours and job tenure requirements, but excludes most self-employed and public sector workers, again, jobs in which Hispanic and black women, respectively, are overrepresented in our sample.

Another hypothesis is that black and Hispanic women are not taking advantage of benefits that are available to them, potentially because of limited awareness. When we restrict to just private sector workers who are most likely to be eligible for CA-PFL, we still find that 39% of black and 38% of Hispanic women report that they received no paid leave through the government, compared to just 14% of white women.

Our finding that black and Hispanic women were significantly less likely to have received help navigating their benefits from anyone, including, most notably, their employers, suggests that women of color may be missing out on benefits that should be available to them because of inadequate information sharing. The extent to which this differential access to information is rooted in occupational segregation, interpersonal racism and discrimination, or some combination of these factors merits further investigation.

Relatedly, it is possible that black and Hispanic women are more fearful of losing their job if they take leave; although we do not have a clear measure of the importance of this concern, prior analysis has shown that indeed fewer black and Hispanic women are in job-protected positions.^54^ Again, the root cause of this differential actual and perceived job security are worthy subjects of future research.

Finally, Hispanic women, many of whom are Spanish speakers, may face language or cultural barriers to accessing information about leave benefits.

The fact that paid leave access depends on the characteristics of one's job suggests that relying on employers to provide this important benefit voluntarily is insufficient for ensuring equity. Government PFML programs are often also similarly deficient, frequently excluding the most economically vulnerable workers, in another manifestation of structural racism.

For example, San Francisco's PPLO provides fully paid leave to eligible workers, but includes only workers employed by private sector employers with at least 20 employees. These restrictions disproportionately exclude low-income workers and completely exclude self-employed and public sector workers.^48^

A more equitable policy approach would resemble Oregon's recently enacted PFML policy, which includes all workers, regardless of firm size or sector, who have earned at least $1000 in wages during a base year and paid into the state's PFML Insurance Fund.^38^

Our study addresses a critical gap in the literature by differentiating the source of paid leave and quantifying the proportion of usual pay received while on leave, thus providing a more complete picture of the extent of inequity in paid leave access. Future studies should attempt to replicate and build on these findings in other settings. We focus exclusively on the San Francisco Bay Area, which is demographically, economically, and politically distinct from other regions. Our relatively small sample of black women, although reflective of San Francisco's demography, limits our power to detect small differences.

Future research should also attempt to further disentangle the mechanisms underlying racial/ethnic disparities in paid leave access, including occupational segregation, policy coverage, fear, awareness, and cultural barriers (particularly for Spanish-speaking women).

## Supplementary Material

Supplemental data

Supplemental data

Supplemental data

Supplemental data

## Abbreviations Used

ACOG: American College of Obstetricians and Gynecologists
CA-PFL: California's paid family leave
EITC: earned income tax credit
FPE: full-pay equivalent
GED: General Education Development
NH: Non-Hispanic
PFML: paid family and medical leave
PPLO: Paid Parental Leave Ordinance

## References

[1] Leonard SA, Main EK, Scott KA, et al. Racial and ethnic disparities in severe maternal morbidity prevalence and trends. Ann Epidemiol. 2019;33:30–36. 30928320 10.1016/j.annepidem.2019.02.007 PMC6502679

[2] Pregnancy Mortality Surveillance System | Maternal and Infant Health | CDC. Published November 25, 2020. Available at www.cdc.gov/reproductivehealth/maternal-mortality/pregnancy-mortality-surveillance-system.htm Accessed December 8, 2020.

[3] Grobman W, Parker C, Willinger M, et al. Racial disparities in adverse pregnancy outcomes and psychosocial stress. Obstet Gynecol. 2018;131:328–335. 29324613 10.1097/AOG.0000000000002441 PMC5785441

[4] Infant Mortality | Maternal and Infant Health | Reproductive Health | CDC. Published September 10, 2020. Available at www.cdc.gov/reproductivehealth/maternalinfanthealth/infantmortality.htm Accessed December 8, 2020.

[5] Beauregard JL. Racial disparities in breastfeeding initiation and duration among U.S. infants born in 2015. MMWR Morb Mortal Wkly Rep. 2019;68:745–748. 31465319 10.15585/mmwr.mm6834a3 PMC6715261

[6] Matoba N, Collins JW. Racial disparity in infant mortality. Semin Perinatol. 2017;41:354–359. 28864275 10.1053/j.semperi.2017.07.003

[7] Collins JW, David RJ, Handler A, et al. Very low birthweight in African American infants: the role of maternal exposure to interpersonal racial discrimination. Am J Public Health. 2004;94:2132–2138. 15569965 10.2105/ajph.94.12.2132 PMC1448603

[8] Gee GC, Ford CL. Structural racism and health inequities. Bois Rev Soc Sci Res Race. 2011;8:115–132. 10.1017/S1742058X11000130 PMC4306458 25632292

[9] Riggan KA, Gilbert A, Allyse MA. Acknowledging and addressing allostatic load in pregnancy care. J Racial Ethn Health Disparities. 2021;8:69–79. 32383045 10.1007/s40615-020-00757-z PMC7647942

[10] Howell EA, Egorova N, Balbierz A, et al. Black-white differences in severe maternal morbidity and site of care. Am J Obstet Gynecol. 2016;214:122.e1–122.e7. 26283457 10.1016/j.ajog.2015.08.019 PMC4698019

[11] Mohapatra S. Black Pain Matters: *The Need for a Health Justice Approach to Chronic Pain Management*. Social Science Research Network, 2016. Available at https://papers.ssrn.com/abstract=2617895 Accessed December 8, 2020.

[12] Attanasio L, Kozhimannil KB. Health care engagement and follow-up after perceived discrimination in maternity care. Med Care. 2017;55:830–833. 28692572 10.1097/MLR.0000000000000773

[13] Andres E, Baird S, Bart J, et al. Maternity leave access and health: a systematic narrative review and conceptual framework development. Matern Child Health J. 2016;20:1178–1192. 26676977 10.1007/s10995-015-1905-9

[14] Aitken Z, Garrett CC, Hewitt B, et al. The maternal health outcomes of paid maternity leave: a systematic review. Soc Sci Med. 2015;130:32–41. 25680101 10.1016/j.socscimed.2015.02.001

[15] Chatterji P, Markowitz S. Family leave after childbirth and the mental health of new mothers. J Ment Health Policy Econ. 2012;15:61–76. 22813939

[16] Tanaka S. Parental leave and child health across OECD countries*. Econ J. 2005;115:7–28.

[17] Baker M, Milligan K. Maternal employment, breastfeeding, and health: evidence from maternity leave mandates. J Health Econ. 2008;27:871–887. 18387682 10.1016/j.jhealeco.2008.02.006

[18] Nandi A, Hajizadeh M, Harper S, et al. Increased duration of paid maternity leave lowers infant mortality in low-and middle-income countries: a quasi-experimental study. PLoS Med. 2016;13:e1001985. 27022926 10.1371/journal.pmed.1001985 PMC4811564

[19] Dagher RK, Dowd BE. Maternity leave duration and postpartum mental and physical health: implications for leave policies. J Health Polit Policy Law. 2014;39:369–416. 24305845 10.1215/03616878-2416247

[20] Hamad R, Modrek S, White JS. Paid family leave effects on breastfeeding: a quasi-experimental study of US policies. Am J Public Health. 2019;109:164–166. 30359107 10.2105/AJPH.2018.304693 PMC6301394

[21] Bullinger LR. The effect of paid family leave on infant and parental health in the United States. J Health Econ. 2019;66:101–116. 31150953 10.1016/j.jhealeco.2019.05.006

[22] Lee BC, Modrek S, White JS, et al. The effect of California's paid family leave policy on parent health: a quasi-experimental study. Soc Sci Med. 2020;251:112915. 32179364 10.1016/j.socscimed.2020.112915 PMC7104658

[23] Rossin M. The effects of maternity leave on children's birth and infant health outcomes in the United States. J Health Econ. 2011;30:221–239. 21300415 10.1016/j.jhealeco.2011.01.005 PMC3698961

[24] Pac J, Bartel A, Ruhm C, et al. Paid Family Leave and Breastfeeding: Evidence from California. Cambridge, MA: National Bureau of Economic Research, 2019. DOI: 10.3386/w25784.

[25] Doran EL, Bartel AP, Ruhm CJ, et al. California's paid family leave law improves maternal psychological health. Soc Sci Med. 2020;256:113003. 32464413 10.1016/j.socscimed.2020.113003

[26] Stearns J. The effects of paid maternity leave: evidence from Temporary Disability Insurance. J Health Econ. 2015;43:85–102. 26218984 10.1016/j.jhealeco.2015.04.005

[27] ACOG Committee Opinion No. 736: Optimizing Postpartum Care. Obstet Gynecol. 2018;131:e140–e150. 29683911 10.1097/AOG.0000000000002633

[28] Murray Horwitz ME, Molina RL, Snowden JM. Postpartum care in the United States—new policies for a new paradigm. N Engl J Med. 2018;379:1691–1693. 30380385 10.1056/NEJMp1806516

[29] Stanczyk AB. Does paid family leave improve household economic security following a birth? Evidence from California. Soc Serv Rev. 2019;93:262–304.

[30] Ybarra M, Stanczyk A, Ha Y. Paid leave, welfare, and material hardship after a birth. Fam Relat. 2019;68:85–103.

[31] Rossin-Slater M, Ruhm CJ, Waldfogel J. The effects of California's paid family leave program on mothers' leave-taking and subsequent labor market outcomes. J Policy Anal Manage. 2013;32:224–245. 23547324 10.1002/pam.21676 PMC3701456

[32] Joshi PK, Geronimo K, Romano B, et al. Integrating racial/ethnic equity into policy assessments to improve child health. Health Aff (Millwood). 2014;33:2222–2229. 25489042 10.1377/hlthaff.2014.1169

[33] Brown S, Herr J, Roy R, et al. Employee and Worksite Perspectives of the Family and Medical Leave Act: Results from the 2018 Surveys. Abt Associates, Inc., 2020, p. 80.

[34] Joshi P, Baldiga M, Earle A, et al. Acevedo-Garcia D. How much would family and medical leave cost workers in the US? Racial/ethnic variation in economic hardship under unpaid and paid policies. Community Work Fam. 2019;1–24.

[35] Choudhury A, Polachek SW. *The Impact of Paid Family Leave on the Timing of Infant Vaccinations*. Social Science Research Network, 2019. Available at https://papers.ssrn.com/abstract=3427622 Accessed February 11, 2020.

[36] Montoya-Williams D, Passarella M, Lorch SA. The impact of paid family leave in the United States on birth outcomes and mortality in the first year of life. Health Serv Res. 2020;55(S2):807–814. 32249413 10.1111/1475-6773.13288 PMC7518811

[37] Klerman JA. Family and Medical Leave in 2012: Technical Report. Final Rep. Published online 2012:174 Accessed May 14, 2019.

[38] National Partnership for Women & Families. *State Paid Family and Medical Leave Insurance Laws*. National Partnership for Women & Families, 2019. Available at https://www.nationalpartnership.org/our-work/resources/economic-justice/paid-leave/state-paid-family-leave-laws.pdf Accessed July 6, 2021.

[39] U.S. Department of Labor, U.S. Bureau of Labor Statistics. National Compensation Survey: Employee Benefits in the United States, March 2019. 2019: p. 549.

[40] Glynn SJ, Farrell J. *Latinos Least Likely to Have Paid Leave or Workplace Flexibility*. 2012. Available at https://www.americanprogress.org/issues/economy/reports/2012/11/20/45394/latinos-least-likely-to-have-paid-leave-or-workplace-flexibility/ Accessed October 30, 2018.

[41] Bartel AP, Kim S, Nam J, et al. *Racial and Ethnic Disparities in Access to and Use of Paid Family and Medical Leave: Evidence from Four Nationally Representative Datasets*. U.S. Bureau of Labor Statistics, 2019. Available at www.bls.gov/opub/mlr/2019/article/racial-and-ethnic-disparities-in-access-to-and-use-of-paid-family-and-medical-leave.htm Accessed June 23, 2020.

[42] Earle A, Joshi P, Geronimo K, et al. *Job Characteristics among Working Parents: Differences by Race, Ethnicity, and Nativity*. U.S. Bureau of Labor Statistics; 2014. Available at https://www.bls.gov/opub/mlr/2014/article/job-characteristics-among-working-parents.htm Accessed December 8, 2020.

[43] Appelbaum E, Milkman R. Leaves That Pay: Employer and Workers Experiences with Paid Family Leave in California. Washington, DC: Center for Economic and Policy Research, 2011.

[44] Shepherd-Banigan M, Bell JF. Paid leave benefits among a national sample of working mothers with infants in the United States. Matern Child Health J. 2014;18:286–295. 23584928 10.1007/s10995-013-1264-3 PMC3840152

[45] Zagorsky JL. Divergent trends in US maternity and paternity leave, 1994–2015. Am J Public Health. 2017;107:460–465. 28103064 10.2105/AJPH.2016.303607 PMC5296693

[46] Slopen M. Type and lengths of family leave among New York City women: exploring the composition of paid and unpaid leave. Matern Child Health J. 2020;24:514–523. 31993933 10.1007/s10995-020-02884-9

[47] Hanks A, Solomon D, Weller CE. Systematic Inequality. Center for American Progress. Available at https://www.americanprogress.org/issues/race/reports/2018/02/21/447051/systematic-inequality/ Accessed December 8, 2020.

[48] Goodman JM, Elser H, Dow WH. Among low-income women in San Francisco, low awareness of paid parental leave benefits inhibits take-up. Health Aff (Millwood). 2020;39:1157–1165. 32634354 10.1377/hlthaff.2020.00157

[49] *The Earned Income Tax Credit, Poverty, And Health*. 2018. Available at https://www.healthaffairs.org/doi/10.1377/hpb20180817.769687 Accessed December 30, 2020.

[50] Evans WN, Garthwaite CL. Giving mom a break: the impact of higher EITC payments on maternal health. Am Econ J Econ Policy. 2014;6:258–290.

[51] Milligan K, Stabile M. Do child tax benefits affect the well-being of children? Evidence from Canadian child benefit expansions. Am Econ J Econ Policy. 2011;3:175–205.

[52] Hoynes H, Miller D, Simon D. Income, the earned income tax credit, and infant health. Am Econ J Econ Policy. 2015;7:172–211.

[53] Hamad R, Rehkopf DH. Poverty and child development: a longitudinal study of the impact of the earned income tax credit. Am J Epidemiol. 2016;183:775–784. 27056961 10.1093/aje/kwv317 PMC4851995

[54] Goodman JM, Dow WH. *Expanded Job Protection Improves Racial and Socioeconomic Equity of Parental Leave Access*. UC Berkeley School of Public Health, 2020. Available at www.populationsciences.berkeley.edu/PPLO Accessed December 30, 2020.

