# Supplementary figures and images for: Racial/Ethnic Inequities in Paid Parental Leave Access
Source: Health Equity. 2021 Oct 13;5(1):738–49. doi: 10.1089/heq.2021.0001 (PMC8665807; doi:10.1089/heq.2021.0001)

Supplementary Figure S1: Duration of maternity leave taken, by race/ethnicity. Notes: *p<0.10; **p<0.05; ***p<0.01


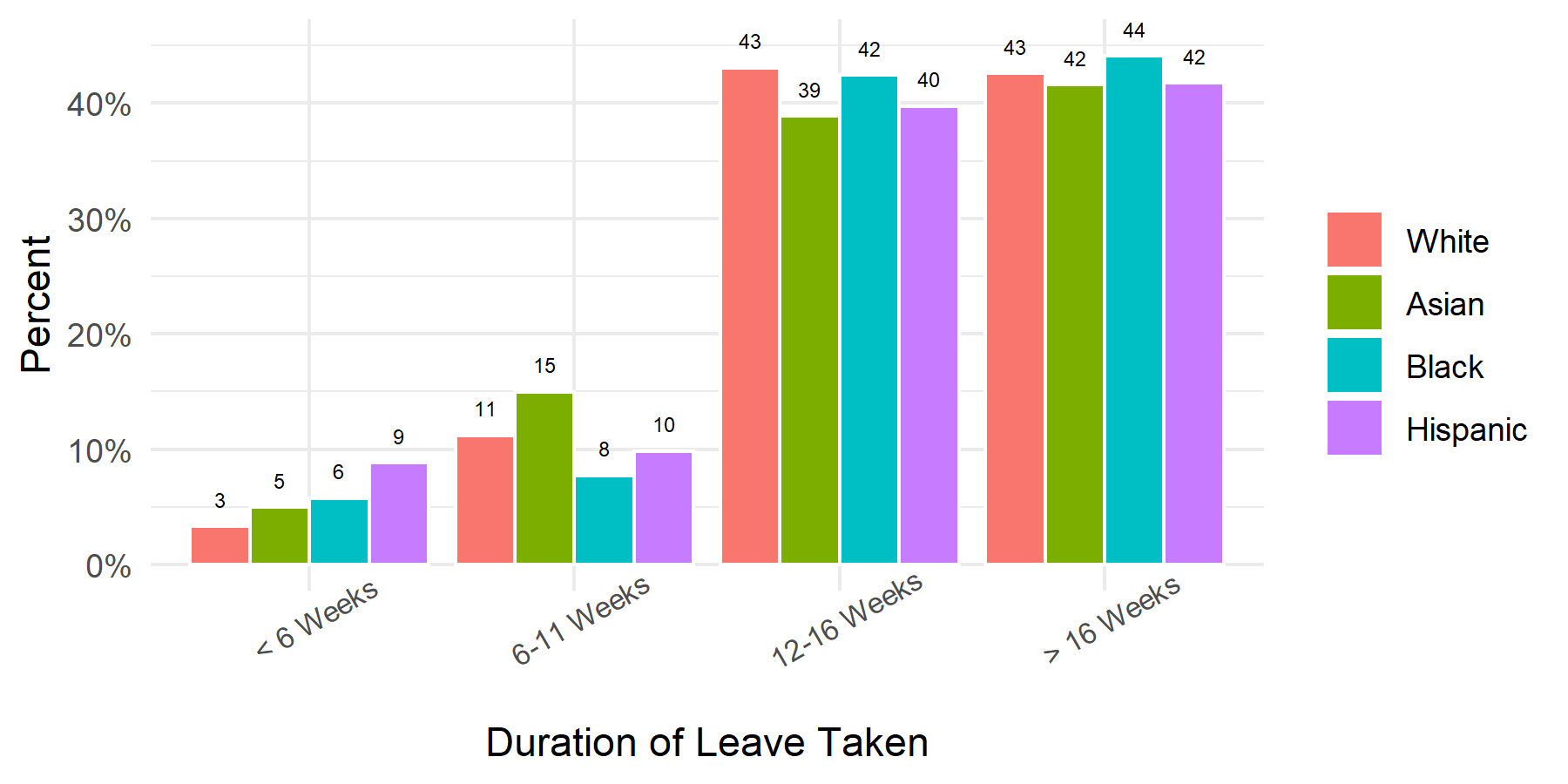

Supplement: Supplemental data [file Supp_FigS1.docx]
