# Supplementary material for: Racial/Ethnic Inequities in Paid Parental Leave Access
Source: Health Equity. 2021 Oct 13;5(1):738–49. doi: 10.1089/heq.2021.0001 (PMC8665807; doi:10.1089/heq.2021.0001)

Supplementary Figure S4: Duration of government- and employer-paid leave among private for-profit workers, by race/ethnicity. Notes: FPE=full-pay equivalent weeks of paid leave. *p<0.10; **p<0.05; ***p<0.01


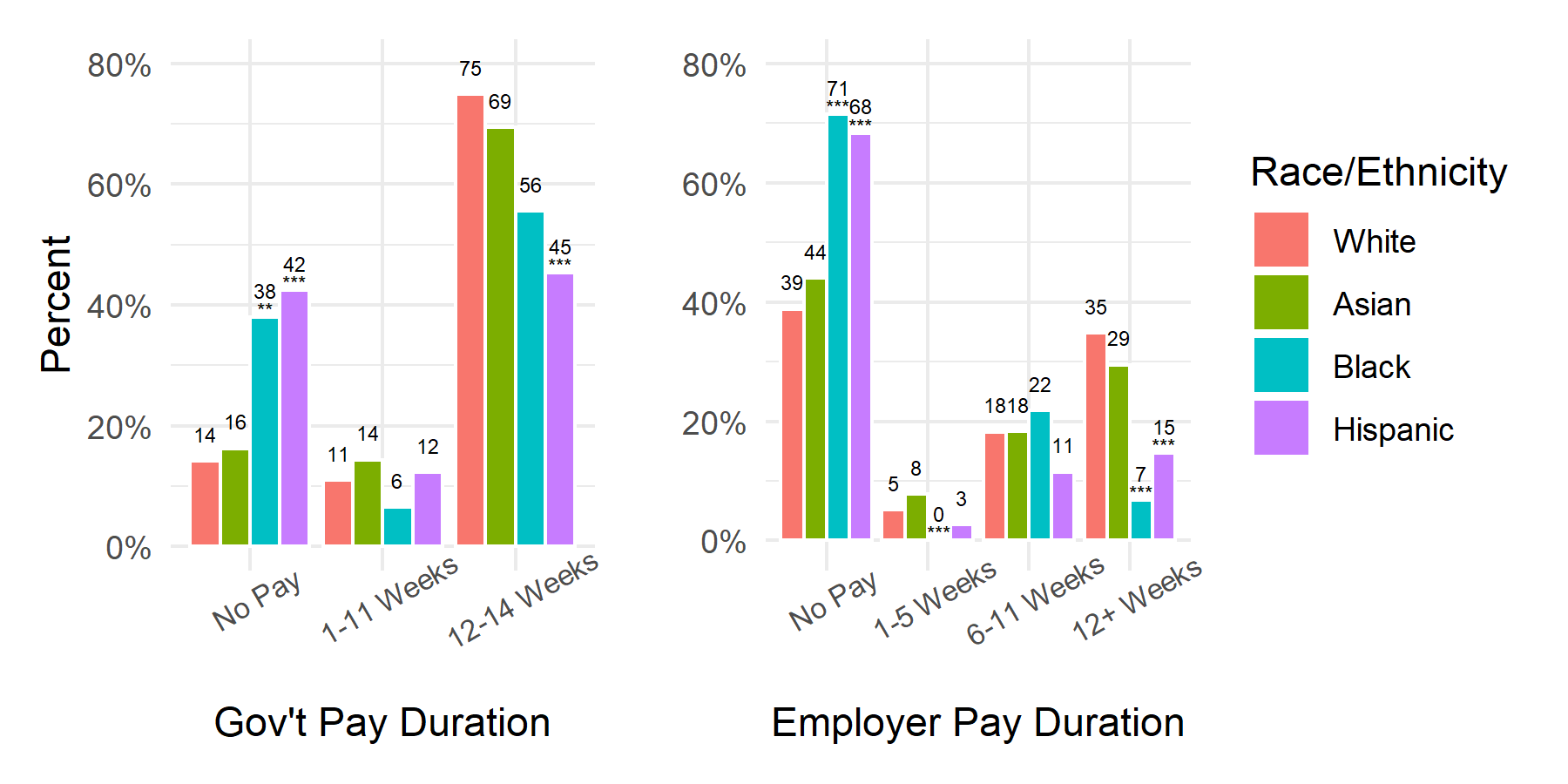

Supplement: Supplemental data [file Supp_FigS4.docx]
